# Synthetic Medicinal Chemistry in Chagas’ Disease: Compounds at The Final Stage of “Hit-To-Lead” Phase

**DOI:** 10.3390/ph3040810

**Published:** 2010-03-25

**Authors:** Hugo Cerecetto, Mercedes González

**Affiliations:** 1 Departamento de Química Orgánica, Facultad de Química, Universidad de la República, Iguá 4225, Montevideo 11400, Uruguay; 2 Laboratorio de Química Orgánica, Instituto de Química Biológica-Facultad de Ciencias, Universidad de la República, Iguá 4225, Montevideo 11400, Uruguay

**Keywords:** Chagas’ disease, “hit-to-lead”, drug-likeness

## Abstract

Chagas’ disease, or American trypanosomosiasis, has been the most relevant illness produced by protozoa in Latin America. Synthetic medicinal chemistry efforts have provided an extensive number of chemodiverse hits at the “active-to-hit” stage. However, only a more limited number of these have been studied *in vivo* in models of Chagas’ disease. Herein, we survey some of the cantidates able to surpass the “hit-to-lead” stage discussing their limitations or merit to enter in clinical trials in the short term.

## 1. Introduction

**Figure 1:**
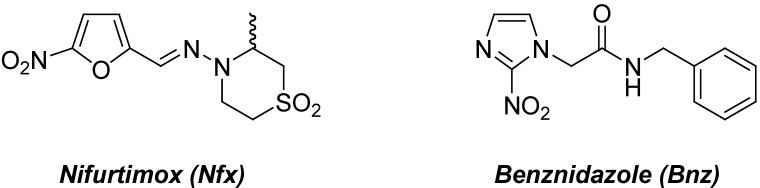
Chemical structures of clinical used drugs for Chagas’ disease.

Despite the fact that Carlos Chagas completely described, near to a century ago [1] the vector, microorganism and clinical signs, American trypanosomiasis, remains the largest parasitic disease burden on the American continent. This illness, also known as Chagas’ disease, is caused by the trypanosomatid parasite *Trypanosoma cruzi* (*T. cruzi*). Like other neglected diseases, it is an important health problem due to inadequate therapy and the lack of an effective vaccine [2]. It has not received much attention by the pharmaceutical industry mainly due to economic considerations. Current clinical treatment of Chagas’ disease relies on two drugs (Figure 1): nifurtimox (Nfx, 3-methyl-4-(5-nitrofurfurylidene-amino)tetrahydro-4*H*-1,4-thiazine-1,1-dioxide, Lampit®, recently discontinued by Bayer) and benznidazole (Bnz, *N*-benzyl-2-(2-nitroimidazolyl)acetamide, Rochagan®, Roche, now produced by LAFEPE, Brazil) discovered empirically more than three decades ago [3]. These drugs are nitroheterocycles that present significant side effects, e.g., Nfx may cause weight loss, skin rash, psychosis, leucopenia, neurotoxicity, peripheral neuropathy, tissue abnormalities, nausea and vomiting; while Bnz may trigger edema, fever, skin rash, peripheral neuropathy, lymphadenopathy, agranulocytosis, thrombocytopenic purpura, articular and muscular pain [4]. The use of these drugs during the acute phase is widely accepted, but their efficacy in the chronic phase is still controversial. Currently, the treatment plan of chronically infected patients is based on the hypothesis that Chagasic cardiomyopathy may be triggered by persistent parasitic infection [5]. Since 2004, the BENEFIT (BENznidazole Evaluation For Interrupting Trypanosomiasis Program) multicenter study involves a randomized, double-blind, placebo-controlled, Bnz clinical trial of 3,000 patients with Chagasic cardiomyopathy from 35 centers of Latin America [6]. Similarly, Nfx, combined with eflornithine, is involved in a multicentre, randomized, open-label, active control phase III trial for treatment of patients with African *Trypanosoma brucei gambiense* trypanosomiasis [7].

Apart from their arguable efficacies in all the disease stages, Nfx y Bnz have showed different efficacies according to both endemic geographical areas and *T. cruzi* strains [8]. However the most relevant problems are their toxic and genotoxic behaviors that convert them into inappropriate drugs for treatment of any kind of disease [4,9,10,11]. Given the unsatisfactory pharmaceutical performance of the currently available drugs, new approaches to specific chemotherapy of Chagas’ disease have been advanced in the last three decades. They will be discussed in the following sections focusing in the synthetic medicinal chemistry and on those compounds at the final stage of the “hit-to-lead” phase and with possibilities of entering the clinical phase.

## 2. Medicinal Chemistry in Chagas’ Disease

Medicinal chemistry, as an interdisciplinary science, has combined all its tools in the discovery of anti-Chagas drugs. Accordingly, efforts have come from biochemistry/molecular biology, computational chemistry, pharmacognosy, pharmacology, drug repositioning, and organic and inorganic chemistry areas. Studies have been done in the different stages of the drug discovery process – hit selection, synthetic development to lead identification, synthetic modifications to lead optimization, and preclinical steps – contributing in a synergistic manner allowing the identification of potential drug candidates.

The information of the complete genome sequences of *T. cruzi* revealed that its genome contains nearly 10,000 protein-coding genes [12]. This vast amount of new information allows the identification of targets in an accurate manner [13,14,15,16,17]. From a medicinal chemistry point of view, several potential biological targets for drugs development have been identified, e.g., geranyltransferase type I, farnesyltransferase, farnesyl pyrophosphate synthase, *trans*-sialidase, cAMP-specific phosphor-diesterases, polyamine and trypanothione synthetic pathways, cystein proteases, glucose 6-phosphate dehydrogenase, glyceraldehyde 3-phosphate dehydrogenase, membrane sterols synthetic pathways, or α-hydroxyacid dehydrogenase, but in some cases their validity and druggability as anti-Chagas targets have been placed in doubt.

**Figure 2:**
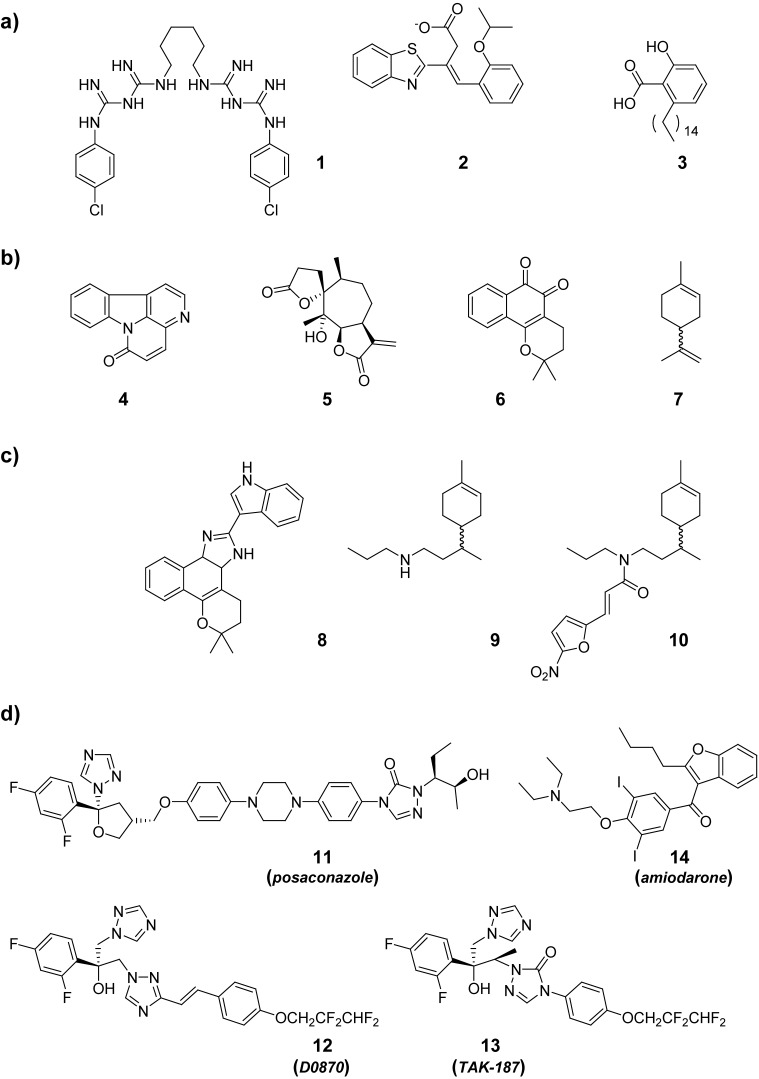
(a) Chemical structures of selected *T. cruzi*-enzymatic inhibitors. (b) Chemical structures of selected natural products with anti-*T. cruzi* activity. (c) Examples of medicinal chemistry based on natural products. (d) Chemical structures of examples of drug-profiling strategy in Chagas’ disease.

Computational chemistry has reinforced the generated *T. cruzi* genomic/proteomic information using tools at the hit selection stage, e.g., virtual screening to identify inhibitors of specific parasite biomolecules [18,19,20], or at the lead optimization step, e.g., developing theoretical models that explain activities [21,22,23]. Figure 2 shows some examples of selected hits with specific enzymatic inhibitory activities.

Latin America vegetation has supplied a great number of active compounds where the pharmacognosts have identified relevant hits to treat Chagas’ disease. Significant leads have come from Argentine, Brazil, Bolivia, Chile, Paraguay and Peru (Figure 2) [24,25,26,27,28,29]. However, scarce examples where medicinal chemistry involve in chemical modifications to attempt improvement of the hits’ activities [29,30,31,32,33].

A great deal of work in the pharmacology/toxicology areas has been published by Argentinean, Brazilian and Chilean research teams. Castro’s group in Argentine has worked on the toxicological profile of the current anti-Chagas drugs, Nfx and Bnz [4], while the Chilean team of Morello has driven aspects related to Nfx’s mechanism of action and improvement of its activity by drug-combination [34,35]. On the other hand, the Brazilian group of de Castro has generated relevant information on experimental chemotherapies for Chagas’ disease working *in vitro* but also *in vivo* (Figure 2c, see below, Section 3) [36,37].

Drug repositioning, or drug profiling, is a medicinal chemistry tool that has also been employed in the lead identification stage for Chagas’ disease drugs. The concept of drug profiling, concerning in the research of either discontinued-, off-patent, or another-application-drug for novel indications, has been developed by Urbina from Venezuela [38]. The concept of the biological redundancy has been successfully applied by Urbina employing well-known antifungal drugs as anti-Chagas agents (Figure 2) [39]. The idea that these drugs have undergone extensive toxicological and pharmacokinetic studies support that their indication as anti-Chagas drugs would involve less risk, cost and time than conventional discovery. Based on previous reports on amiodarone’s (**14**, Figure 2) *in vitro* antifungal activity [40], Urbina found that this drug, used as an antiarrhythmic in Chagasic cardiomyopathy, also possess a synergic anti-*T. cruzi* effect when it is co-administered together with the antifungal posaconazole (compound **11**, Figure 2) [41].

Organic and inorganic medicinal chemistry, mainly from academic centers and collaborative networks, has contributed with relevant information from the design, the synthesis, the structural modifications optimizing identified-hits, and the structure-activity relationships. Some of these results and approaches will be discussed in the following section describing those agents emerge from the “active-to-hit” stage.

### 2.1. Compounds from the “Active-To-Hit” Phase

The different synthetic medicinal chemistry approaches, at the “active-to-hit” development stage, come mainly from Argentine, Brazil, Germany, Spain, United States, United Kingdom, Uruguay, and Venezuela academic partnerships, in some cases sponsored by WHO and DNDi [42]. The anti-*T. cruzi in vitro* studies have been described against three different forms of the parasite, the vectorial epimastigote-, the bloodstream trypomastigote-, and the intracellular amastigote-forms. 

**Figure 3:**
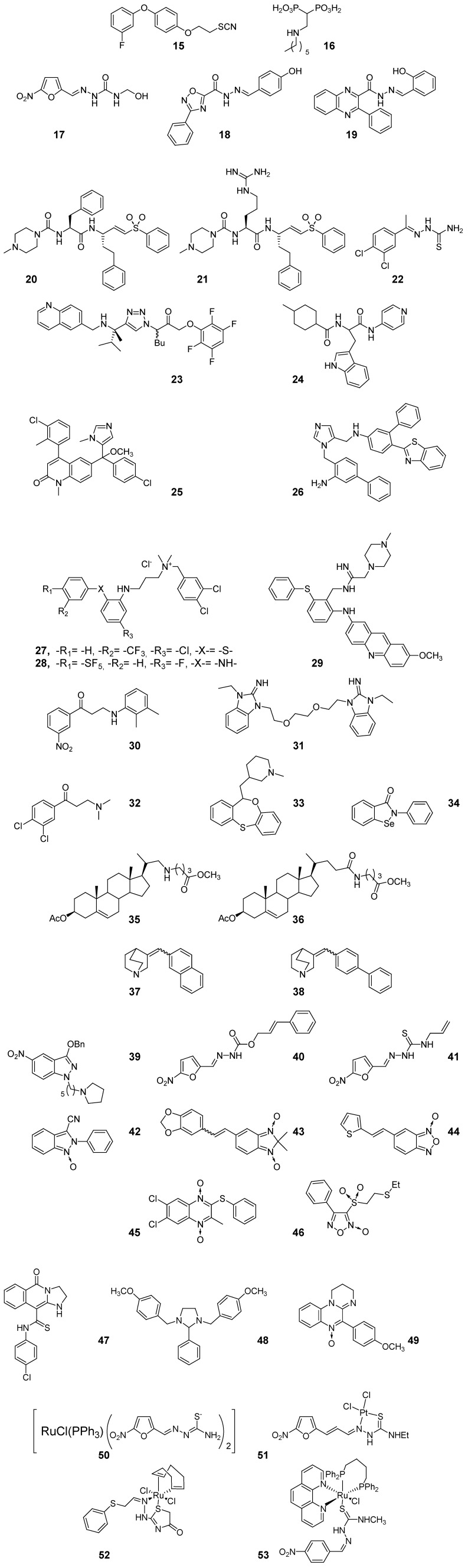
Examples of anti-*T. cruzi* products emerging from the synthetic medicinal chemistry, hitherto at the “active-to-hit” development phase.

Inhibition of enzymes involved in the parasites’ sterol syntheses pathway have been studied by Rodríguez’ team, from Argentine, who have worked on two different chemical systems, thiocyanates and bisphosphonic acids. Compound **15** (Figure 3), a thiocyanate derivative [43,44,45,46], a hit with micromolar anti-amastigote activity acts by reduction of the parasite endogenous sterols as result of the inhibition of squalene synthase. The bisphosphonic acids have been developed as inhibitors of *T. cruzi* farnesyl diphosphate synthase (TcFPPS), resulting compound **16** (Figure 3) a molecular prototype with nanomolar anti-amastigote activity and adequate activity against TcFPPS [47,48].

In Brazil, organic chemistry teams have been working on the identification of new molecular hits with capability to inhibit cruzipain, the major cysteine protease from *T. cruzi* [22,23,49,50]. Ferreira’s group has identified the nitrofurazone derivative **17** (Figure 3), initially developed as a synthetic prodrug intermediate, with good anti-*T. cruzi* activity and besides with cruzipain-inhibition property [51]. This compound shows lack of mutagenic property which make it as a good candidate for “hit-to-lead” studies (see below, Section 3.3). Recently, both Leite’s and Lima’s teams have described *N-*acyl-hydrazones, e.g., **18** and **19** (Figure 3), with good *in vitro* activities against the whole parasite, trypomastigote and epimastigote forms, and, according to the theoretical calculations, with potential ability to inhibit the desired enzyme [52,53].

Using cruzipain as the biological target, the group of the Sandler Center ( United States) has described the study of an important number of chemical-compound libraries identifying agents with optimal cruzain-inhibition capability [54]. Compound **20**, belonging to the vinyl-sulfone family, and its guanidinyl analogue **21,** named as K777 and WRR483 [55,56,57] respectively (Figure 3), have been identified as hits with excellent *in vitro* activity against whole parasite and enzyme that led them to be considered for *in vivo* studies (see below, Section 3). Thiosemicarbazones and 1,2,3-triazoles, e.g., hits **22** and **23** (Figure 3), are other groups of cruzipain inhibitor-pharmacophores studied by these researchers [58,59]. Whereas **23** completely eradicated amastigote *T. cruzi* in cultures thiosemicarbazone **22** had only modest anti-proliferative *T. cruzi* activity.

Additionally, McKerrow’s team have worked on the research and development of *T. cruzi* cytochrome P450 CYP51 (sterol 14α-demethylase) inhibitors finding the hit **24** [60] (Figure 3) that acts as enzymatic inhibitor and also as anti-amastigote agent with low to no toxicity against the studied mammal systems [61]. In the same sense Gelb’s group, from Washington University (United States) has been studying *T. cruzi* CYP51 inhibitors using as chemical template the human protein farnesyltransferase inhibitor tipifarnib. Unexpectedly, this compound with excellent *in vitro* anti-*T. cruzi* activity, was also a *T. cruzi* CYP51 inhibitor [62] allowing the design, syntheses and biological studies of new series of derivatives with activity against this enzyme. The lead compound, **25** (Figure 3), displayed picomolar activity against cultured *T. cruzi*. Interesting hits have been identified from this group being imidazole **26** (Figure 3) submitted to *in vivo* studies (see below, Section 3) [63,64,65].

The trypanothione-based thiol metabolism has also been used as target for new anti-*T. cruzi* drug identification [66]. In this sense, Dr. Krauth-Siegel’s team, from Heidelberg University (Germany) working together with partnerships from Argentine, France, Switzerland, United Kingdom, United States, and Uruguay, has studied a great number of compounds as trypanothione reductase (TR) inhibitors. The most recent hits described by Krauth-Siegel *et al.* as TR inhibitors are **27–29** (Figure 3) [67,68,69].

Fairlamb’s group, from United Kingdom, sponsored by DNDi and with the participation of partnerships from Australia, Brazil, Canada, Cuba, France, Spain, Switzerland, United States, and Venezuela, has looked forward for new molecular prototypes with ability to inhibit *T. cruzi* TR. Some of the most recent hit compounds are **30–34** (Figure 3). None of them were evaluated against the entire parasite [70,71,72,73].

Also from United Kingdom, the Gilbert-group’s chemical efforts working on the anti-*T. cruzi* “active-to-hit” stage should be mentioned. With the participation of partnerships from Australia, Brazil, Canada, Cuba, France, Spain, Switzerland, United States, and Venezuela, Gilbert’s synthetic approaches (e.g., **35–38**, Figure 3) have involved inhibitors of *T. cruzi* dihydrofolate reductase [74], azasterols as inhibitors of sterol 24(25)-methyltransferase of *T. cruzi* [75,76,77,78], and quinuclidines as inhibitors of squalene synthase of *T. cruzi* [79,80,81]. The best anti-*T. cruzi* quinuclidines developed by Gilbert *et al.* had an effect on the sterol composition of *Leishmania mexicana* promastigotes (e.g., **37** and **38**, Figure 3).

Our group, from Universidad de la República (Uruguay) together with partnerships from Argentine, Brazil, Chile, Italy, Spain, and Venezuela has described different kind of molecular prototypes mainly in the field of the intraparasite-free radical-releasing agents. Some of the most relevant examples are the nitroheterocycles **39–41** [82,83,84,85], the *N-*oxides **42–46** [86,87,88,89,90,91], and some other interesting heterocycles **47–49** [53,92,93,94]. Some of them have passed to the “hit-to-,lead” phase (see below, Section 3) and others, according to their biological behavior, should advance to this stage in the short to medium-term.

Inorganic synthetic medicinal chemistry has also provided very interesting structural motives as hits for anti-*T. cruzi* drugs. Some of the first examples have came from the Sánchez-Delgado team, from Venezuela [95,96], however currently Brazilian and Uruguayan groups have been reporting the bulk of results related to inorganic strategies. Different metals have been reacted with chemical entities with antiparasitic/enzymatic inhibitory activity (e.g., **50–52**, Figure 3) [97,98,99,100,101,102] and without specific biological properties (e.g., **53**, Figure 3) [103]. These compounds have demonstrated to act by at least two mechanisms [104,105,106] and some of them have been advanced to *in vivo* studies (see below, Section 3).

## 3. Compounds at the Final Stage of “Hit-to-Lead” Phase

A significant number of candidates, from the “active-to-hit” phase, have been submitted to some of the subsequent studies corresponding to the “hit-to-lead” stage. The compounds will be described according to the laboratory and country origins, and aspects related to their *drug-like* properties –e.g., oral bioavailability, toxicity, mutagenicity- will be analyzed and discussed.

**Figure 4:**
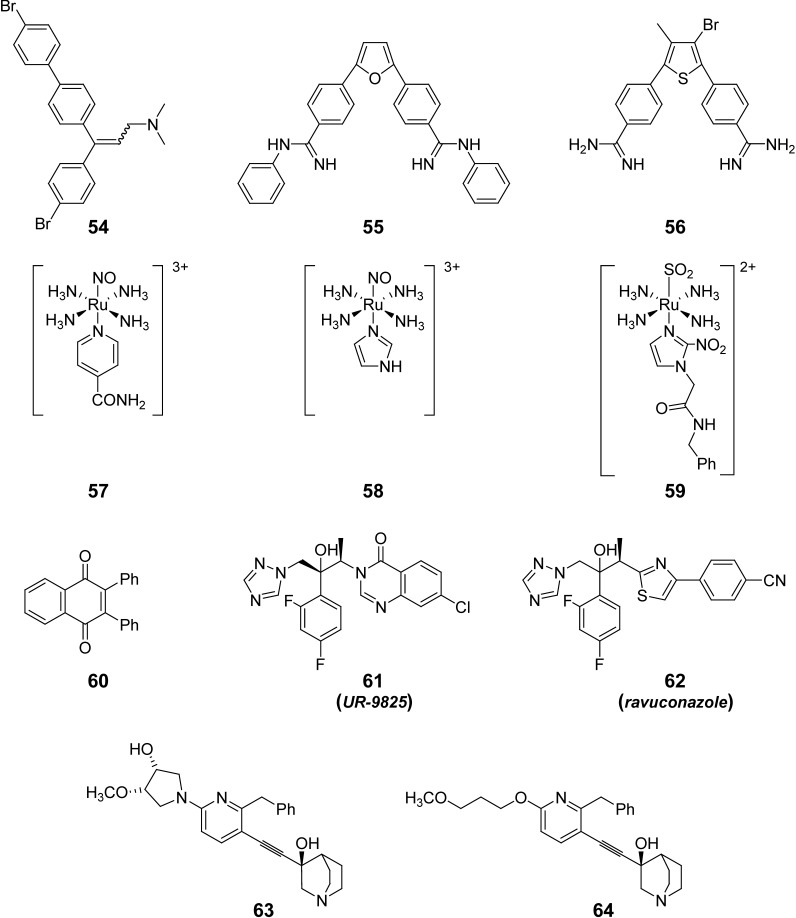
Examples of anti-*T. cruzi* products emerging from the synthetic medicinal chemistry, hitherto at the “hit-to-lead” development phase.

From the Laboratório de Biologia Celular-Instituto Oswaldo Cruz in Brazil, the ergosterol inhibitor compound **54** (as a geometric isomers-mixture, Figure 4) has been studied in a model of infected animals (oral administration of 5 mg/kg b.w. for nine consecutive days, starting on the day of inoculation). This compound produced a consistent suppression of parasitemia combined with full protection against death, 100% of animals’ survival at the end of the assay *vs* 75% for Bnz-treated animals (at 100 mg/kg b. w.) [107,108]. On the other hand, the diamidines **55** and **56** (Figure 4), active *in vitro* against the different forms of the parasite, have been studied *in vivo* using different drug-administration schedules by i.p. route [109,110,111]. Compound **55** reduced cardiac parasite load and down-modulated the expression of CD8^+^ T cells in the heart tissues that revert the electrocardiography alterations in acute and chronic infection, leading to an increase in the animals’ survival rates. The best results with diamidine **56 **were obtained when the animals received 25 mg/kg b.w. in two intervals, one at the onset and the second at the parasitemia peak, with 100% of animals’ survival, reduction of the cardiac parasitism although only a reduction of 40% in the parasitemia levels. These classes of compounds deserve further investigation, for example pharmacokinetic properties and metabolic stability, based on the adequate anti-*T. cruzi in vivo* results.

From University of São Paulo, Brazil, Franco’s team has worked in the inorganic medicinal chemistry field evaluating *in vivo* ruthenium complexes **57**, **58** and **59** (Figure 4) [112,113]. Complexes **57** and **58** have been developed as NO-releasing compounds and they probed to be active* in vitro* against the parasite while *in vivo* results, i.p. administration of 100 nmol of each compound/kg b.w. daily for 15 consecutive days, showed that they were discrete in both the decreasing of the parasitemia and the animals’ survival rates with decreasing the occurrence of myocarditis (mainly for **58**). Complex **59**, synthesized using imidazole-nitrogen from Bnz as coordinating atom, has been evaluated *in vivo* for its toxicity -LD_50_ using *up-and-down* test protocol- and for its trypanonosomicidal activity in a murine model of acute Chagas’ disease. The antiparasitic studies were done using different dosage’ schedules, e.g., oral and i.p. administration and doses of 100 nmol/kg b.w./day or 385 nmol/kg b.w./day, at least 1,000-fold lower than LD_50_, for 15 consecutive days or for only three days, the fifth, sixth, and seventh days that precede the parasitaemic peak. Compound **59** proved to protect the infected animals eliminating, according to the micrographs, the hearts and skeletal muscles amastigotes. This compound is hydrosoluble but no information about pharmacokinetic properties and metabolic stability was mentioned.

Maldonado’s laboratory, from The University of Texas (United States) has evaluated *in vivo* the competitive inhibitor of *T. cruzi* lipoamide dehydrogenase **60** (Figure 4) [114,115]. The naphthoquinone was administered 24 hours post-infection -10 mg/kg b.w./ initially one daily i.p. dose for 3 consecutive days, and then 2 more doses, with an interval of 1 day between the doses- observing depletion in parasitemia and animals’ survival up to 60% at 70 days post-infection without apparent signs of toxicity on the uninfected treated animals.

Compounds **20** and **21** (K777 and WRR483, respectively, Figure 3), from Sandler Center (United States) have been analyzed in different models of acute Chagas’ disease, *i.e.* mice and dogs [116,117,118]. For example, when **20** was administered orally at 50 mg/kg b. w. twice daily for 14 days, to infected dogs the myocardial damage was ameliorated according to serum cardiac troponin I levels and histopathology findings. This compound, which is water soluble, orally bioavailable (20%), neither toxic nor mutagenic [119] and produces an additive effect on parasite killing when used in combination with Bnz [54], has now entered formal preclinical drug development investigations [54]. On the other hand, the vinyl-sulfone **21**, an analog of the parent **20** contains a protonizable arginine side-chain that turn it into a better cruzipain inhibitor with activity against the parasite in culture and in an animal model of disease [54].

Compound **26** (Figure 3), from the synthetic medicinal chemistry projects at the University of Washington (United States) had the best *in vivo* profile when it was administered orally, at 20 or 50 mg/kg b.w. twice per day for 21 days, to mice in an acute model of Chagas’ disease [65]. From the pharmacokinetic studies the authors have not observed significant **26** losses out to 5 h, showing its mouse plasma stability. The animals tolerated the drug treatments without apparent side effects however studies of toxicity/mutagenicity were not done. Although the *in vivo* activity of **26 **was lower than that of posaconazole (**11**, Figure 2), its synthesis is much simpler and its potential cost of goods lower.

Urbina, initially from Instituto Venezolano de Investigaciones Científicas (Venezuela) and now at the University of Cincinnati in the United States, has worked* in vivo* with well-known ergosterol biosynthesis inhibitors. The antifungals terbinafine, ketoconazole or itraconazole displayed suppressive, but not curative, effects against *T. cruzi* infections in animal models of Chagas’ disease [120]. However, triazoles (**11–13**, Figure 2; **61** and **62**, Figure 4) selective inhibitors of fungal and protozoan cytochrome P-450-dependent CYP51 were potent inducing radical parasitological cure in murine models of acute and chronic Chagas’ disease [121,122,123,124,125,126,127]. The remarkable *in vivo* antiparasitic activities of some of them result from a combination of their potent and selective intrinsic anti-*T. cruzi* activity and special pharmacokinetic properties (long terminal half-life and large volumes of distribution). For example, posaconazole, **11**, has terminal phase half-life of 7–9 h in mice and rats [128]. On the other hand, azole **61** has very short half-life in mice but working in a canine model demonstrated curative activity against established infections of the virulent Y strain of *T. cruzi* with very low toxicity but drug resistance was encountered with the Berenice-78 strain [39]. Another example of inadequate pharmacokinetic properties in mice was evidenced in the treatment with **62** however these results do not necessarily rule out the potential usefulness of this compound in the treatment of human *T. cruzi* infections since its activity is very high. According to Urbina, triazoles **11** and **62** would be entering into the clinical studies for the treatment of human chronic Chagas disease in the next 12 months [39] (http://www.dndi.org/press-releases/532-eisai-and-dndi-enter-into-a-collaboration.html) moreover taking in account that **11** has been demonstrated to be active in a case of a Chagas’ disease patient compromised with systemic lupus erythematosus [129].

Another interesting group of compounds that have been studied *in vivo* by Urbina are the quinuclidine derivatives (e.g., **63** and **64**, Figure 4) [130] under development for cholesterol control in humans. The *in vivo* behavior of compound **63** resulted better than that of **64** producing animal survival rates of 100% and arresting development of parasitemia in the studied murine model of acute disease and in one of the assayed condition, oral treatment at a concentration of 50 mg/kg of b. w./day for 30 consecutive days. The better antiparasitic properties of **63** could be due to its better pharmacokinetic properties, with lower clearance rate in rats than **64** and wide tissue distribution, such as heart, spleen, liver, and instestines [130].

Others examples that should be mentioned for their *in vivo* relevance are the approaches with products isolated from natural resources. One example comes from Paraguay [25] where canthin-6-one (**4**, Figure 2), isolated from *Zanthoxylum chiloperone* as well as the total alkaloids extract from stem bark led parasitological clearance in the experimental conditions, oral or subcutaneal administration at 5mg/kg b. w./day for 2 weeks for the pure product and 50mg/kg b. w./day for 2 weeks for alkaloidal extract. Another example is the sesquiterpene lactone **5** (Figure 2) isolated from *Ambrosia tenuifolia* Sprengel (Asteraceae) that decreased the parasitemia levels during the experiment and promoted an animals’ survival rate of 100% when it was administered i.p. at 1 mg/kg b. w./day for five consecutive days [26].

**Figure 5:**
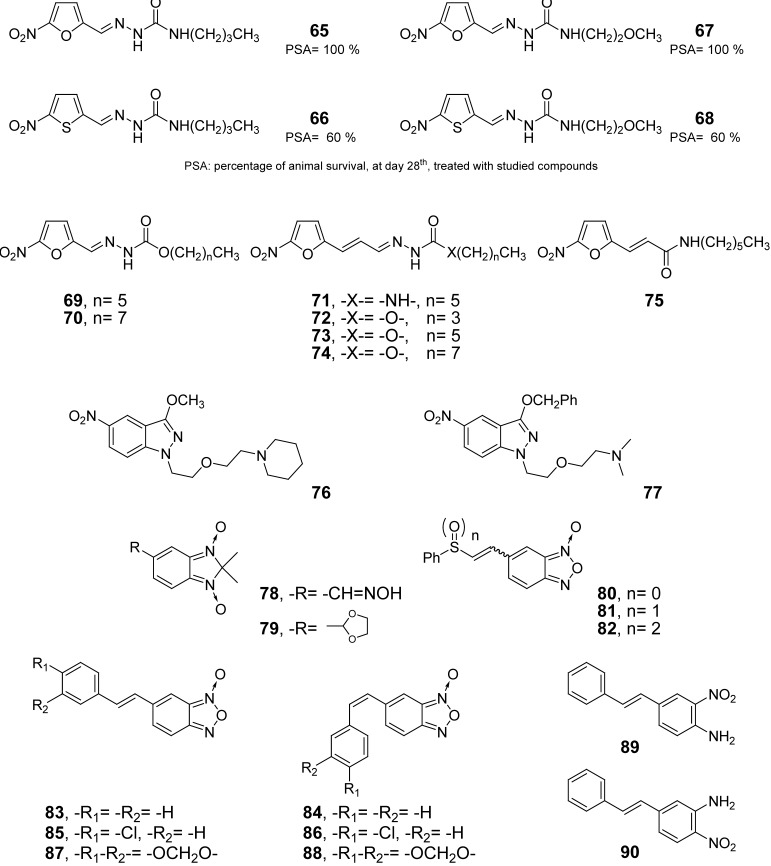
Examples of anti-*T. cruzi* products hitherto at the “hit-to-lead” development phase from our laboratory.

Our group at Universidad de la República (Uruguay), in collaboration with Argentinean and Paraguayan teams has assessed nineteen nitroheterocyclic derivatives and ten *N-*oxide derivatives in different murine models of acute Chagas’ disease [131,132,133,134,135,136]. In our first approach, we found that each 5-nitrofuran derivative was more *in vivo* active than the corresponding 5-nitrothiophene analogue, e.g., comparing animals’ survival rates of derivatives **65**, **66**, **67** and **68** (Figure 5) where they were administered orally during 10 days at 66 mg/kg b. w./day. After that, a series of ten 5-nitrofurans were tested oral and intraperitoneally. Based on the toxicity on murine models investigated with a single administration of 7.5- and 6-fold the therapeutic dose (60 mg/kg b. w.) we selected three among the new derivatives (**71**, **74**, and **75** Figure 5) for testing *in vivo* anti-*T. cruzi* activities. A clear relationship between chemical structure and both acute toxicity and *in vivo* anti-*T. cruzi* activity was observed, showing that derivatives **65**, **67**, **69**, and **70** promoted higher toxicity, in healthy animals, than derivatives **71–73** and carbazate **74** (Figure 5) was lesser *in vivo* anti-trypomastigote agent than the semicarbazones **65**, **67**, and **71**, or the amide **75 **(Figure 5). Derivative **71** showed an excellent anti-*T. cruzi* profile for the smooth muscle with adequate profile in the cardiac one. Unfortunately, derivatives **71**, **74**, and **75** have demonstrated mutagenicity in the Ames test (against *S. Typhimurium* TA 98) with and without S9 fraction (unpublished data). The 5-nitroindazoles **76** and **77** were selected, for their lack of *in vitro* unspecific cytotoxicity, to be evaluated in a murine model of acute Chagas’ disease, oral administration at 30 mg/kg b. w./day for 10 days (using Bnz as positive control at 50 mg/kg b. w./day for 10 days). Taking into account that the doses of the tested compounds were very different, 78 mmol/kg b. w./day for **76** and **77** and 192 mmol/ kg b. w./day for Bnz, compound **76** showed better *in vivo* performance than the reference drug, Bnz, in animals’ survival rates and anti-*T. cruzi* antibody levels. On the other hand, according to parasitemia, animals’ survival rates and antibodies levels compound **77** have an *in vivo* behavior as good as Bnz. Although further structural modifications and deeper pharmaceutical studies have been done [137,138], this class of compounds deserves further investigation based on the adequate anti-*T. cruzi in vivo* results. The same experimental protocol has been used to evaluate the benzimidazole di-*N*-oxides **78** and **79** (Figure 5) where both compounds were able to rescue mice from death and lowered anti-*T. cruzi* antibodies levels, especially **78**, with only 10 doses in a short-term scheme. Regrettably, compound **78** has demonstrated mutagenicity in the Ames test (against *S. Typhimurium* TA 98) in absence of metabolic activation (unpublished data). Pharmacokinetic and metabolic-stability studies for compounds **65–77** were not performed.

Benzofuroxans **80–82** (as geometric isomer mixtures, Figure 5), developed as *T. cruzi* cruzipain inhibitors, have been evaluated in an acute model of Chagas’ disease, oral administration at 60 mg/kg b. w./day for 30 days (6 day treatment, 1 day rest, until 30 doses completed). The mixture of isomers **81** has the best *in vivo* profile among the three tested benzofuroxans and better than Bnz considering the antibody levels. Furthermore, no signs of toxicity were observed in the studied animals, during the treatment, and none of the animals treated with **80–82** died during the treatment while in the control group the survival rate was 75%. Following the same experimental protocol, and as DNDi project, benzofuroxans **83–88 **(Figure 5), and the geometric isomers-mixtures, have been studied *in vivo* against different *T. cruzi* strains, e.g., Tulahuen 2 and Colombiana (resistant to Nifurtimox and Benznidazole) strains, and two wild strains, one isolated from the wild reservoir *Didelphis marsupialis* and another one from a Uruguayan patient. The results showed that **83** and the equimolecular mixture **83**:**84** were able to reduce the parasite loads of animals with fully established *T. cruzi* infections with, in some cases, comparable results to that obtained with both Nfx and Bnz. The results of histological studies indicated that they could be adequate agents in preventing inflammatory infiltrates and tissue damage, particularly in heart of infected animals. The main problem found in these studies was the low compound solubility in the chosen vehicles. Furthermore, according to the acute-toxicity study administration of compound **83**, at high doses, did not produce remarkable damage on the animals. As part of the research project supported by DNDi organization others preclinical studies with **83–88**, e.g., scaling-up and analytical procedures, *in vitro* metabolism and toxicity, have been performed [139,140,141,142,143]. The mutagenic studies have indicated that benzofuroxans **81**, **82** (data not published), **83**, **85–88** are mutagenic, in some cases with metabolic activation, like Nfx and Bnz. Derivatives **80**, **84** and the equimolecular mixture **83**:**84** were not-mutagenic in both conditions, unfortunately the isomer **84** is metabolized *in vitro*, and probably *in vivo*, to **83 **by hepatocyte microsomal and cytosolic fractions. In deep studies of the metabolites responsible of mutagenicity for derivative **83**, one of the main metabolite, **89**, and not the other, **90**, is mutagenic with S9 activation.

### 3.1. Drug-like Properties of the anti-T. cruzi compounds at the Ending Stage of “Hit-to-Lead” Phase

Adequate *drug-like* properties of one lead compound allow the researchers to convert it into a successful drug. The term *drug-like* became used following the work of Lipinski *et al.* [144] who examined the structural properties that affect the physicochemical properties of solubility and permeability and their effect on drug absorption. On the other hand, toxicity studies were traditionally performed during the clinical development phase however it has been a major cause of drug candidate attrition during preclinical and clinical phases. For this reason, the early knowledge about toxicity candidate also allows the researchers to become it in a successful drug.

In this stage of the studies and as a form to anticipate the potential use as drugs, the compounds described here (Section 3) are analyzed in their *drug-like* properties – e.g., oral bioavailability, genotoxicity, mutagenicity – using *in silico* approaches (Table 1). For compounds’ oral bioavailability Lipinski’ and Veber’ [145] rules are employed, extracting properties from the free websites http://www.molinspiration.com/ and http://www.organic-chemistry.org/prog/peo/, while for the compounds’ potential toxicity/mutagenicity *in silico* classifications are extracted from the website http://www.organic-chemistry.org/prog/peo/ or using the free software Toxtree-v1.60 (http://toxtree.sourceforge.net/). From http://www.organic-chemistry.org/prog/peo/ the descriptors *drug-likeness* and *drug-score* are extracted. According to these predictive tools (which represent only some of the free-available ones), one could determine the most promissory candidates that should behave adequately in the next in depth preclinical studies (see colored cells in Table 1): 1) the allylamine **54** and the diamidine **56** with expected both satisfactory oral biodisposal and lack of toxicity; 2) the quinuclidines **63** and **64** with the best values of *drug-score* and *drug-likeness*, according to the classification of the *Osiris Properties Explorer* tools (http://www.organic-chemistry.org/prog/peo/), but with reservations in the potential genotoxic effects (micronucleus assay in rodents) according to *Toxtree* software; 3) the benzimidazole di-*N*-oxides **78** and **79** with, except for *drug-score* values, similar results that quinuclidine derivatives. Unfortunately, the *in vitro* mutagenicity of compound **78** has been confirmed. Taking in account the behaviors of Nfx and Bnz in these tools, together with the experimental data about their drug-properties, other compounds put on Table 1 could be considered in profound preclinical trials, e.g., **4**, **13**, **55**, **61**, **67**, **68**, **69**, **76**, **77**, **83**, **84**, **87**, and **88**.

**Table 1:** Drug-like properties of compounds described in Section 3.

Compd.	Meets Lipinski’s rule	Meets Veber’s rule	*Drug-likeness*^1^	*Drug-score*^1^	Toxic effects^1^	Alerts for mn^2^	Carcinogenic - mutagenic effects^2^
**4**	y (0)^3^	y (0)^3^	-0.984	0.54^5^	n^6^	I^7^	A^8^
**5**	y (0)	y (0)	-10.47	0.22	y (m,i)	I	C
**11**	n (2)	n (1)	4.51	0.05	y (m,t,i,r)	I	C
**12**	y (1)	n (1)	-9.42	0.16	y (i,r)	I	C
**13**	y (1)	y (0)	-4.34	0.13	y (i,r)	I	C
**20**	y (1)	n (1)	0.48	0.29	y (i)	I	C
**21**	n (3)	n (2)	1.21	0.41	y (i)	I	C
**26**	n (2)	y (0)	-2.68	0.05	y (m,t,i)	I	A,F
**54^10^**	y (1)	y (0)	-3.08	0.14	n	II	C
**55**	y (1)	y (0)	0.38	0.28	n	II	C,D
**56**	y (1)	y (0)	-2.61	0.29	n	II	C
**57**	y (1)	n (1)	-^9^	-	-	I	C
**58**	y (1)	n (1)	-	-	-	I	C
**59**	n (2)	n (1)	-	-	-	I	C
**60**	y (1)	y (0)	-1.38	0.23	y (r)	I	A
**61**	y (0)	y (0)	3.41	0.51	y (r)	I	C
**62**	y (0)	y (0)	-7.2	0.08	y (m,t,i,r)	I	C
**63**	y (0)	y (0)	2.76	0.64	n	I	C
**64**	y (0)	y (0)	1.43	0.53	n	I	C
**65**	y (0)	y (0)	1.01	0.55	y (m)	I	A
**66**	y (0)^3^	y (0)^3^	-2.61^4^	0.33^5^	y (m)^6^	I^7^	A^8^
			1.57	0.81	n		
**67**	y (0)	y (0)				I	A
**68**	y (0)	y (0)	-2.09	0.49	n	I	A
**69**	y (0)	y (0)	-15.56	0.19	y (i)	I	A
**70**	y (0)	n (1)	-23.57	0.21	y (i)	I	A
**71**	y (0)	y (0)	-17.57	0.11	y (m,i)	I	A
**72**	y (0)	y (0)	-8.91	0.17	y (m,i)	I	A
**73**	y (0)	y (0)	-17.57	0.11	y (m,i)	I	A
**74**	y (0)	n (1)	-25.58	0.11	y (m,i)	I	A
**75**	y (0)	y (0)	-13.95	0.23	y (m)	I	A
**76**	y (0)	y (0)	-5.98	0.43	n	I	A
**77**	y (0)	y (0)	-9.05	0.40	n	I	A
**78**	y (0)	y (0)	-2.43	0.53	n	I	C
**79**	y (0)	y (0)	-8.82	0.49	n	I	C
**80^10^**	y (0)	y (0)	-3.64	0.24	n	I	A
**81^10^**	y (0)	y (0)	-5.44	0.32	n	I	A
**82^10^**	y (0)	y (0)	-12.28	0.25	y (i)	I	A
**83**	y (0)	y (0)	-3.53	0.28	n	I	A
**84**	y (0)	y (0)	-3.53	0.28	n	I	A
**85**	y (1)	y (0)	-0.95	0.22	y (m)	I	A/B
**86**	y (1)	y (0)	-0.95	0.22	y (m)	I	A/B
**87**	y (0)	y (0)	-1.48	0.27	n	I	A
**88**	y (0)	y (0)	-1.48	0.27	n	I	A
**Nfx**	y (0)	y (0)	0.65	0.16	n (m,t,r)	I	A
**Bnz**	y (0)	y (0)	-3.32	0.18	n (m,r)	I	A

^1^ From http://www.organic-chemistry.org/prog/peo/. ^2^ According to Toxtree-v1.60 software [146,147]. mn: micronucleus. ^3 ^y: adjust; n: no adjust; in parenthesis number of violations of the rule. ^4^ Positive value is for a compound that contains predominatly fragments frequent in commercial drugs. ^5 ^The drug score combines *drug-likeness*, cLogP, logS, MW and toxicity risks. ^6 ^y: toxic effect, mutagenic (m), tumorigenic (t), irritant (i); reproductive effective (r); n: non toxic effects. ^7^ Structure alerts for the in vivo micronucleus assay in rodents. I: At least one positive structural alert for the micronucleus assay; II: No alerts for the micronucleus assay. ^8^ Benigni/Bossa rulebase for mutagenicity and carcinogenicity; A: structural alert for genotoxic carcinogenicity; B: structural alert for nongenotoxic carcinogenicity; C: no alerts for carcinogenic activity; D: potential* S. typhimurium* TA100 mutagen based on QSAR; E: unlikely to be a* S. typhimurium* TA100 mutagen based on QSAR; F: potential carcinogen based on QSAR; G: unlikely to be a carcinogen based on QSAR; H: for a better assessment a QSAR calculation could be applied; J: error when applying the decision tree. ^9^ “-”: no result. ^10^ Study as *E*-isomer. The color of the cell refers to: green, good result; yellow, intermedium; rose, bad result.

### 3.2. Scale-Up Information of the anti-T. cruzi Compounds at the Ending Stage of “Hit-to-Lead” Phase

Another relevant aspect in the preclinical and clinical stage, and in the pharmaceutical production, is the adequate product scalability in the synthesis or extraction. This means both environmental friendly processes and Good Manufacturing Practices (GMP). In the case of Chagas’ disease, a neglected disease of deprived countries, low costs are also essential.

Only for some of the compounds described here (Section 3) the scale-up procedures have been assayed [54,139] besides the antifungal azoles (**11–13**, **61**, and **62**) and the cholesterol-control quinuclidines (**63** and **64**). On the other hand, these compounds with at least one chiral center, together with **20** and **21**, or those with geometric isomers, like **54**, **57–59**, **71–75**, and **80–88**, have the additional complexity that specific and well-controlled synthetic procedures must be used because only one of the stereoisomers is the responsible for the activity. In this sense, compound **26** is a good alternative, as it is less expensive to produce as claimed by the researchers, than other *T. cruzi* CYP51 inhibitors [65].

### 3.3. Candidates to be Submitted at the “Hit-to-Lead” Phase in the Short to Medium-Term

**Figure 6:**
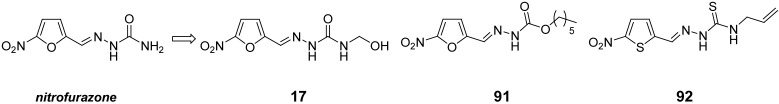
5-Nitrofuran and 5-nitrothiophene derivatives without mutagenic effects (Ames test).

We want to highlight three compounds whose lack of mutagenicity makes them potential candidates that should be submitted to the “hit-to-lead” phase in the short to medium-term. One of them is 5-nitrofuran **17** (Figure 3 and Figure 6), that has been developed by Ferreira, from Universidade do São Paulo ( Brazil), as nitrofurazone prodrug intermediate. Besides its excellent anti-*T. cruzi* activity it demonstrated lower mutagenic effects than the parent compound nitrofurazone [148,149]. The other two compounds are the 5-nitrofuran and 5-nitrothiophene, **91** and **92**, respectively (Figure 6), developed in our group [85,150]. In addition to their excellent *in vitro* anti-*T. cruzi* activities they were non-mutagenic in both conditions with and without metabolic activation (unpublished data).

## 4. Conclusions

In conclusion, there is currently a number of candidates for preclinical and clinical trials for the identification of new effective anti-Chagas’ drugs, guaranteeing tolerability, safety profile, and selectivity. Another relevant aspect that should be studied and considered is the cost of production of the new agents that could guarantee patient access to these drugs.
